# Evidence-based journalism

**DOI:** 10.3325/cmj.2011.52.212

**Published:** 2011-04

**Authors:** Farrokh Habibzadeh, Mahboobeh Yadollahie

In their daily practice, editors face different problems and questions. To handle these situations, there are many policies and guidelines established by well-known influential organizations such as World Association of Medical Editors, International Committee of Medical Journal Editors, Committee on Publication Ethics, Council of Science Editors, European Association of Science Editors, and Eastern Mediterranean Association of Medical Editors, to name only a few.

Like in other fields, many of regulations set by these editors’ organizations originate from our eminent predecessors. Many of such claims are mainly based on common sense and are supported by no solid evidence. As an example, one of the very basic rules that most of editors abide by and teach the authors about is that a good article title should be concise (1-3). However, we found that articles with longer titles receive more citations than those with shorter titles (4). This example reveals that the validity of a statement should not be judged based merely on the name and fame of its author. This is a clear example of fallacy of defective induction; in informal logic also termed as ‘appeal to authority,’ which was mainly discussed by the English philosopher and physician John Locke (5). Had no one doubted the well-known statements made by eminent scholars, the Earth would have been flat and stood still in the center of the universe; it would have been circumnavigated by all the celestial bodies including the Sun (6); solar eclipse would have been the evidence of Gods’ wrath; heavy objects would have fallen faster than light objects; and psychotic patients should have been treated by exorcists, to give only a few examples. All these underline the fact that, like in other scientific disciplines, research is of paramount importance in the field of journalism and editor’s craft.

Dr Stephen P. Lock, the former Editor of the *BMJ*, was the first who coined the term “journalology” to describe the application of bibliometrics to journals’ evaluation (7). Since several years ago, we have witnessed various congresses presenting results of studies conducted in this field. An example of such congresses is the International Congress on Peer Review and Biomedical Publication held every four years with the aim of improving the quality and credibility of peer review in biomedical journals (8). There are also many articles published on this issue. But, are all the findings obtained from such research studies applicable to all journals and editors?

In 1991, Dr Gordon H. Guyatt of McMaster University, described the concept of evidence-based medicine (9,10). Through this approach, we search and evaluate the current evidence and determine if the best available evidence obtained from research studies is applicable to our patient. Soon, the new paradigm opened its way into many disciplines. I believe the principles of evidence-based practice can also be used in the field of journalism. To demonstrate how it might work, let’s solve a problem through an example.

Many reports revealed that blinding reviewers to authors’ identity does neither significantly improve nor worsen the quality of peer review (11,12). Masking a manuscript is indeed very difficult, time consuming, and expensive, particularly if you want to do that completely, if it is possible at all. Can all editors (say those working in a small scientific community) send the submitted manuscripts for peer review without blinding reviewers to the identity of authors?

One of the basic approaches used in evidence-based practice is termed PICO, which stands for the *P*opulation, the *I*ntervention, the *C*omparison, and the *O*utcome (13). Here, we want to explore whether single-blind review (where the reviewers know the authors but the authors do not know the reviewers [*I*nvestigation]) is as good as double-blind review (neither the reviewers nor the authors know each other [*C*omparison]) in a small scientific community (*P*opulation). The primary *O*utcome is to measure if the quality of the reviews increased and the secondary *O*utcome is to measure if the process is done with lower cost. Obviously, since our *P*opulation of reviewers (working in a small scientific community) is not similar to the *P*opulation of reviewers studied by the two aforementioned studies (working in large scientific communities) (11,12), we cannot be quite sure about how well the results apply to our setting.

We have to stop saying “*magister dixit”* and instead should only rely on the results obtained from standard research methods. However, not all research findings are useful for all settings. Employing the basic concepts of evidence-based practice we have to examine the current body of evidence to see if it is applicable to our setting or not. Perhaps, this is a time to extend the concepts of evidence-based practice to the field of journalism – a time for a paradigm shift from eminence-based to evidence-based journalism. We can take the universally accepted standards, customize them according to our own needs, culture, and setting and give feedback to the world. In this way, biomedical journalism, the most important aim of which is to improve human health and life, can progress in different parts of the world.

Evidence-Based Medicine Working GroupEvidence-based medicine: A new approach to teaching the practice of medicine.JAMA19922682420510.1001/jama.268.17.24201404801
